# *rbims*: an R package for integrative functional profiling and pathway-level discrimination in metagenome-assembled genomes

**DOI:** 10.3389/fbinf.2026.1831383

**Published:** 2026-07-13

**Authors:** Karla P. López-Martínez, Stephanie Hereira-Pacheco, Diana Hernández-Oaxaca, Frida López-Ruiz, Mirna Vázquez-Rosas-Landa

**Affiliations:** 1 Facultad de Ciencias, Universidad Nacional Autónoma de México, Mexico City, Mexico; 2 Laboratorio de Interacciones Bióticas, Centro de Investigación en Ciencias Biológicas, Universidad Autónoma de Tlaxcala, Tlaxcala, Mexico; 3 Red de Estudios Moleculares Avanzados, Campus III, Instituto de Ecología A.C., Xalapa, Veracruz, Mexico; 4 Universidad Autónoma Metropolitana, Unidad Xochimilco, Mexico City, Mexico; 5 Unidad Académica de Ecología y Biodiversidad Acuática, Instituto de Ciencias del Mar y Limnología, Universidad Nacional Autónoma de México, Mexico City, Mexico

**Keywords:** functional profiling, hydrocarbon degradation, metagenome-assembled genomes, metagenomics, pathway-level analysis, R package

## Abstract

Metagenomics enables the recovery of metagenome-assembled genomes (MAGs), providing access to the metabolic potential of uncultured microbial communities that drive ecosystem function and biogeochemical cycles. However, as MAGs datasets increase in size and complexity, comparing functional repertoires and identifying ecologically meaningful traits across experimental gradients becomes increasingly difficult. Here, we present *rbims*, a modular R package for integrative functional profiling of MAGs and metagenomic datasets. *rbims* supports annotations from KEGG, dbCAN, InterProScan, MEROPS, and PICRUSt2, and enables the calculation of gene presence/absence, raw abundance, and pathway coverage, as well as metadata-informed comparative analyses and publication-ready visualizations. Beyond descriptive profiling, rbims implements an exploratory discriminant framework that combines compositional differential analysis (ALDEx2) with random forest–based feature ranking to prioritize candidate metabolic traits associated with environmental factors. Importantly, it extends gene-level analysis to pathway-level directional bias testing, allowing users to evaluate whether the majority of genes within a metabolic route are consistently enriched toward a given condition. We applied *rbims* to 42 MAGs recovered from a hydrocarbon enrichment experiment in the North Atlantic Ocean. The workflow identified widespread hexadecane and phenanthrene degradation potential, detected enriched oxidoreductase-related protein families, and revealed a strong pathway-level directional bias toward deep-water MAGs for phenanthrene, naphthalene, and hexadecane degradation pathways. By integrating annotation parsing, quantitative trait analysis, statistical discrimination, and visualization in a reproducible framework, rbims provides a user-friendly platform for functional interpretation in genome-resolved metagenomics.

## Introduction

Prokaryotes represent the most abundant and metabolically diverse life forms on Earth, playing central roles in global biogeochemical cycles and ecosystem stability (Oren, 2004). In marine environments, microbial densities can reach 10^4^–10^6^ cells per milliliter, generating immense genetic and functional diversity (Whitman et al., 1998). These communities regulate nutrient turnover, carbon fluxes, and energy transfer, and respond rapidly to environmental perturbations such as temperature shifts, oxygen depletion, pollution, and hydrocarbon exposure (Atlas, 1998; Doney et al., 2012; Porter and Feig, 1980; Whitman et al., 1998). Despite their ecological relevance, linking genomic potential to ecosystem-level function remains a major challenge in microbial ecology.

Metagenomics enables the reconstruction of microbial genomes directly from environmental samples through metagenome binning, producing metagenome-assembled genomes (MAGs) (Dick et al., 2009; Kang et al., 2015). These draft genomes provide access to the metabolic potential of uncultured microorganisms and allow genome-resolved ecological analysis (Imelfort et al., 2014; Hugerth et al., 2015). However, modern studies routinely generate dozens to thousands of MAGs, making it increasingly difficult to systematically compare functional repertoires, quantify metabolic traits, and extract biologically meaningful patterns across experimental gradients.

Several tools support functional profiling of microbial genomes, including METABOLIC (Zhou et al., 2022), MEBS (Anda et al., 2017), and KEGG Decoder (Graham et al., 2018), which provide comprehensive metabolic reconstructions but are primarily command-line-based and offer limited integration with metadata-driven statistical analysis. Within the R ecosystem, packages such as Thanos (Zhao et al., 2024) and MegaR (Dhungel et al., 2021) facilitate functional exploration but focus either on pathway-centric summaries or phenotype prediction, often relying on assumptions, such as stable gene copy numbers across taxa. Importantly, few tools provide an integrated statistical framework that moves beyond presence/absence or coverage summaries towards quantitative, factor-aware discrimination of metabolic traits.

Here, we present *rbims*, a modular and flexible R package designed to integrate functional annotation parsing, quantitative trait analysis, and statistically informed pathway interpretation in genome-resolved metagenomics. *rbims* supports annotations generated from multiple databases and tools, including KEGG (Kanehisa and Goto, 2000), dbCAN (Zheng et al., 2023), InterProScan (Zdobnov and Apweiler, 2001), MEROPS (Rawlings, Tolle, and Barret, 2004) and PICRUSt2 (Langille et al., 2013), allowing users to consolidate heterogeneous outputs into a unified analytical framework. The package enables calculation of gene presence/absence, raw abundance, and pathway coverage, and supports integration of genome- or sample-level metadata for stratified comparisons across experimental factors. Beyond descriptive summaries, rbims implements an exploratory discriminant analytical framework that combines compositional differential analysis via ALDEx2 with random forest–based feature ranking to prioritize candidate metabolic traits associated with environmental gradients (Fernandes et al., 2013). In this framework, random forest is used as an exploratory ranking step rather than as a predictive classifier, while statistical interpretation relies primarily on compositional effect sizes and pathway-level directional bias testing. Importantly, rbims extends gene-level discrimination to pathway-level directional bias analysis, testing whether the majority of genes within a given metabolic route are consistently enriched toward a specific experimental condition. This approach allows users to distinguish between isolated gene variations and structurally coherent pathway-level shifts. In its current implementation, rbims treats pathways as curated sets of genes or functional identifiers and is intended for functional profiling and pathway-level interpretation rather than network modeling or graph-based inference. In addition, *rbims* includes customizable bubble plots and heatmaps for publication-ready visualization, as well as a curated internal database of aerobic and anaerobic hydrocarbon degradation pathways not fully represented in KEGG. To illustrate its utility, we applied *rbims* to MAGs recovered from a hydrocarbon enrichment experiment in the North Atlantic Ocean (Vázquez Rosas Landa et al., 2023), revealing detailed metabolic patterns associated with hexadecane, phenanthrene, and naphthalene degradation across depth gradients.

## Methods

### Implementation and workflow

**FIGURE 1:**
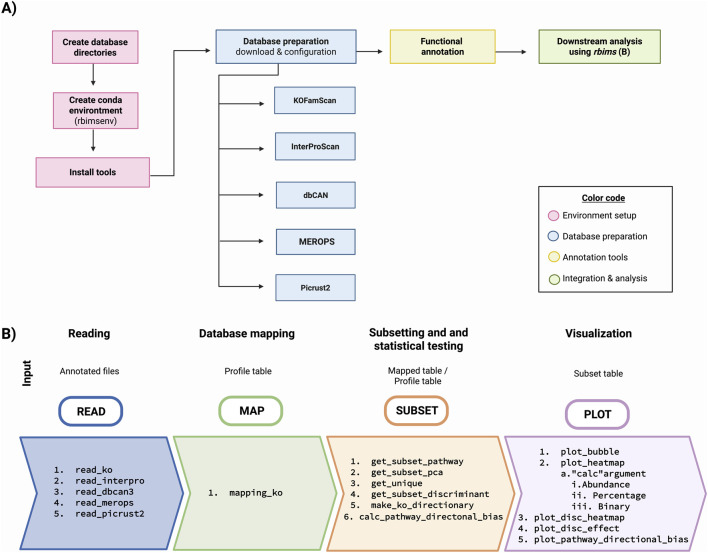
Overview of the rbims workflow. **(A)** Steps to create the *rbims* conda environment (rbimsenv). It integrates external tools (from KEGG, InterProScan, dbCAN, MEROPS, and Picrust2) to run proteome annotations and it creates outputs ready to use by *rbims*. **(B)** Workflow in R. In blue, functions to import annotations (read_*()). In green, function to map profile tables (mapping_ko()). In orange, functions to extract traits via get_subset_*() and calc_pathway_directional_bias(). In purple, functions to visualize the results (plot_*()). *rbims* can read any or all annotations from external tools, and although it can be run within the conda environment, it is not dependent on it.

The *rbims* package consists of four modules that support the exploration of metabolic potential in metagenome-assembled genomes (MAGs): (1) functional annotation via external tools, (2) import of annotation outputs into R, (3) functional analysis, (4) basic visualization and export of results (Figure 1).

### Functional annotation (external tools)

*rbims* allows users to analyze and visualize their own functional annotations as long as they were produced under specific guidelines to match the *rbims* read functions. To solve potential inconsistencies while reading the outputs, we offer a conda environment, rbimsenv (See data and code availability) that integrates annotation tools such as KEGG via KofamScan v1.3.0 (Kanehisa and Goto, 2000), dbCAN v4.1.4 (Zheng et al., 2023), InterProScan v5 (Zdobnov and Apweiler, 2001), and MEROPS database (Rawlings et al., 2004) via BLASTp v2.17.0 (Camacho et al., 2009). Once the annotations are generated, users can import them into R for analysis with *rbims* (Figure 1A).

### Importing annotations into R

*rbims* includes dedicated functions to parse the database outputs: read_ko(), read_dbcan(), read_merops(), and read_interpro(). These functions extract the frequency of functional identifiers per genome and format them as standardized data frames. A fifth function, read_picrust2(), allows for importing KO-based predictions for *metabarcoding* data from PICRUSt2 (Douglas et al., 2020), making them compatible with other *rbims* tools. Each function includes an optional write argument to export the resulting tables (Figure 1B).

### Functional analysis

The core *rbims* functions analyze the frequency tables obtained from the annotations. For KEGG-based data, the mapping_ko() function retrieves pathway information using Bioconductor’s KEGGREST package and the DiTing cycles tool (Tenenbaum, 2024; Xue et al., 2021). Additional functions, such as get_subset_pathway() or get_subset_unique(), allow users to extract relevant subsets based on biological questions (e.g., “which MAGs encode specific hydrocarbon pathways?”). Users can perform three types of calculations: Binary: presence or absence of a gene or function (1 if x > 0; 0 if x = 0), Abundance: raw gene counts per function (∑x), Coverage: percentage of genes recovered for a pathway (x/k) × 100, where x is the number of detected genes and k is the total genes in the reference pathway (Figure 1B). These calculation options are summarized in Table 1.

**TABLE 1:** Summary of calculation options available in rbims. Where *x* = number of detected genes in a MAG, and *k* = total genes defined for a pathway in the reference database.

Metric	Description	Formula
Binary	Presence/Absence per function	1 if *x* > 0, 0 if *x* = 0
Abundance	Raw counts of hits per function	∑ *x* (number of genes)
Percentage (coverage)	Percent of genes recovered for a pathway	(*x*/*k*) × 100

**TABLE 2:** Functional comparison of rbims with related tools. It assesses comparisons in downstream functional profiling, pathway exploration, and metabolic interpretation.

Feature	*rbims*	Thanos	MetQy	MetaPath	MNet/mmnet
Main input	Functional annotation tables from MAGs/metagenomes	MAGs or contigs with depth information and target genes/HMMs	KEGG genes, genomes, modules, or user-defined gene sets	Metagenomic abundance mapped to metabolic reactions	Metabolic or omics-derived network data
KEGG compatibility	Yes	Yes	Yes	Yes	Yes/pathway-network dependent
Support for PFAM/dbCAN/MEROPS/PICRUSt2 outputs	Yes	No/limited	No	No	No/limited
Feature abundance calculation	Yes; from multiple annotation outputs	Yes; based on depth scores	Limited; query/completeness focused	Reaction/pathway abundance	Depends on input
Pathway coverage or completeness	Yes; pathway coverage	Yes; pathway-level aggregation	Yes; module completeness fraction	No; focuses on significant subnetworks	Yes; pathway/network enrichment
Metadata stratification	Yes	Yes/via phyloseq-style metadata	Limited/downstream	Group comparison only	Limited/downstream
Multivariate analysis	Yes; PCA-oriented exploration	No/downstream	PCA-related summaries	No	Network-based summaries
Differential or enrichment analysis	Yes; ALDEx2-based discrimination and pathway directional bias	No/downstream	No/downstream	Yes; differential subnetwork analysis	Yes; enrichment-oriented
Pathway directional bias test	Yes	No	No	No	No
Built-in/exportable visualizations	Yes	Yes	Yes	Limited/output-oriented	Yes/partial

To identify metabolic traits associated with experimental factors, *rbims* implements a discriminant analytical framework through the function get_subset_discriminant(), operating at two hierarchical levels: gene-level (KO-level) and pathway-level. At the gene level, get_subset_discriminant() takes as input a matrix of KEGG Ortholog (KO) counts per genome (or sample), where rows represent KOs and columns represent MAGs. Counts are analyzed using the ALDEx2 package (Fernandes et al., 2013), which applies a centered log-ratio (CLR) transformation to account for the compositional structure of count data. Effect sizes are calculated as the median difference in CLR-transformed abundances between experimental groups. Positive effect values indicate enrichment toward the second level of the grouping factor, whereas negative values indicate enrichment toward the first level. In parallel, *rbims* applies a random forest-based feature-ranking step to the same KO abundance matrix. This step is used to prioritize candidate KOs based on their contribution to group separation and is interpreted as an exploratory ranking procedure rather than a validated predictive classifier. rbims integrates ALDEx2-derived effect sizes and random forest importance scores into a consensus table that prioritizes candidate discriminant KOs (Figure 1B).

To evaluate the stability of the random forest feature-ranking step, we repeated the random forest analysis across resampled datasets. In each iteration, MAGs were sampled within each depth group to preserve group structure, a random forest classifier was trained on the resampled KO abundance matrix, and KOs were ranked according to MeanDecreaseGini importance. We then calculated the frequency with which each KO appeared among the top-ranked features across iterations. This analysis assessed the stability of feature prioritization, not the predictive classification performance.

To extend interpretation beyond individual genes, *rbims* enables a pathway-level directional bias analysis based on the discriminant output. KOs are first linked to curated metabolic routes using make_ko_dictionary(), and pathway-level aggregation is performed with calc_pathway_directional_bias(). For each pathway containing *n* KOs, the number of KOs with positive effect values (x) is calculated. Under the null hypothesis that gene-level effects are symmetrically distributed between groups (expected proportion = 0.5), an exact binomial test is applied to evaluate whether the observed proportion (x/n) significantly deviates from random expectation. Two-sided Clopper–Pearson confidence intervals are computed for the estimated proportion, and p-values are adjusted using false discovery rate (FDR) correction. This hierarchical framework allows *rbims* to distinguish between isolated gene-level differences and structurally coherent pathway-level enrichment across experimental gradients (Figure 1B). Unlike conventional enrichment or overrepresentation tests, this analysis does not ask whether a pathway contains more significant genes than expected. Instead, it evaluates whether the genes assigned to a pathway show a coherent direction of change toward the same experimental condition.

### Basic visualization

*rbims* provides publication-ready visualization functions tailored to different analytical stages. For exploratory and coverage-based analyses, plot_bubble() and plot_heatmap() summarize gene or pathway presence, abundance, and coverage across genomes, with optional metadata stratification (Figure 1B).

For discriminant analyses, plot_disc_heatmap() visualizes the distribution of selected KOs across experimental groups, highlighting patterns of functional representation, while plot_disc_effect() displays effect size estimates and statistical support, facilitating interpretation of directional enrichment. Additionally, pathway-level results from the directional bias analysis can be visualized using plot_pathway_directional_bias(), which summarizes the proportion of positively enriched KOs per pathway together with confidence intervals and adjusted significance values (Figure 1B).

### Internal hydrocarbon database

**FIGURE 2:**
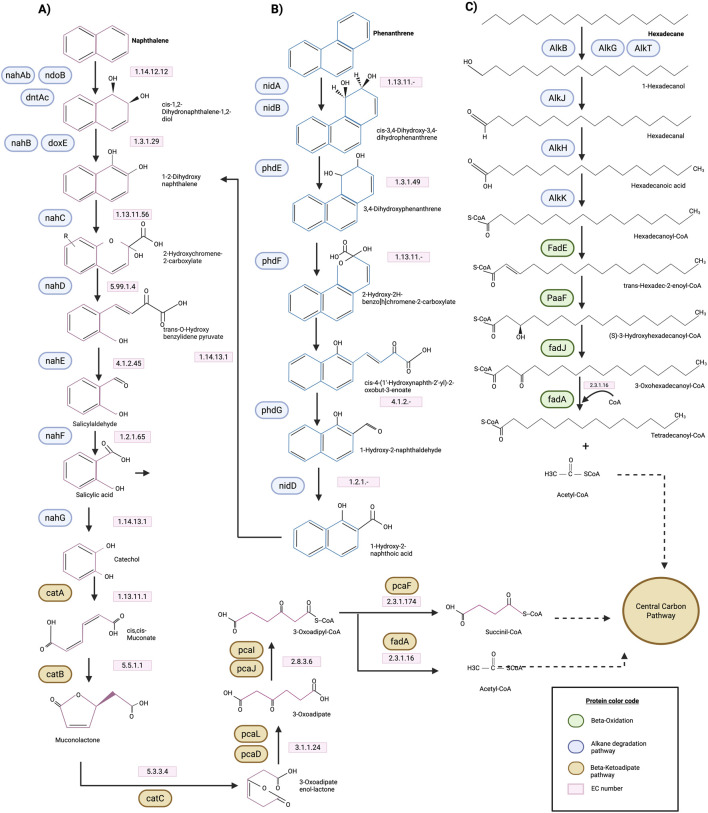
Main metabolic pathways associated with hydrocarbon degradation in the rbims database. The diagram shows the main hydrocarbon degradation pathways in the *rbims* database and the genes involved in each step. The three pathways correspond to the degradation of **(A)** naphthalene, **(B)** phenanthrene, and **(C)** hexadecane, which converge through beta-oxidation into the central carbon metabolism. Colors indicate different pathway categories: blue represents alkane degradation, and green shows beta-oxidation steps. Pink boxes indicate the enzyme commission (EC) numbers associated with each reaction.

*rbims* includes a curated internal database of aerobic and anaerobic hydrocarbon degradation pathways (Figure 2). These include pathways for compounds such as hexadecane, naphthalene, and phenanthrene, which are not covered in KEGG or DiTing Cycles (Xue et al., 2021). The database is indexed by KEGG Ortholog (KO) identifiers and can be queried using functions like get_subset_pathway() in combination with mapping_ko() outputs.

## Results

### Hydrocarbon pathways exploration

We used *rbims* to analyze 42 metagenome-assembled genomes (MAGs) recovered from a hexadecane DNA-based stable-isotope probing experiment conducted in the North Atlantic Ocean (Vázquez Rosas Landa et al., 2023). Protein-coding genes were predicted with Prodigal v2.6.3 and functionally annotated using KofamScan and InterProScan v5.31–70.0 within the *rbims* conda environment. KEGG Ortholog (KO) annotations were imported into R using read_ko() and mapped with mapping_ko() to KEGG modules, pathways, genes, and enzymes, as well as to biogeochemical cycles from the DiTing database and the internal rbims_pathways database (Supplementary Table S1) (Aramaki et al., 2020; Hyatt et al., 2010; Zdobnov and Apweiler, 2001).

**FIGURE 3:**
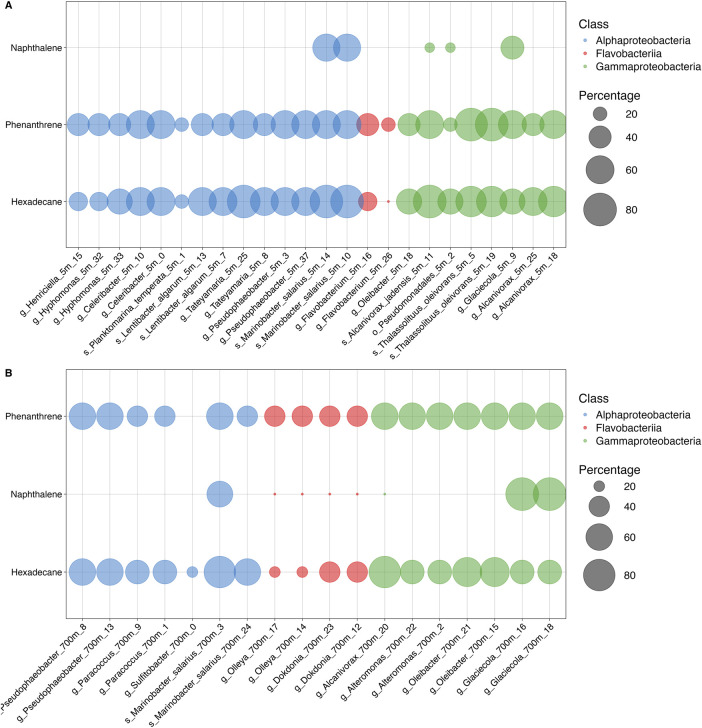
Bubble plot showing coverage of hydrocarbon degradation pathways across MAGs. **(A)** Percentage coverage of the hexadecane degradation pathway (HDP), phenanthrene degradation pathway (PDP), and Naphthalene degradation pathways (NPP) in MAGs from the 5 m depth sample. **(B)** Same coverage metrics in MAGs from the 700 m depth sample. Each bubble represents a MAG and is positioned by genus. Bubble size reflects pathway coverage based on KO presence; color indicates taxonomic affiliation.

To explore hydrocarbon degradation potential, we focused on three pathways curated in the internal “rbims_pathways” database: hexadecane degradation (HDP), phenanthrene degradation (PDP), and naphthalene degradation (NPP). These pathways were compiled from published descriptions of aerobic hydrocarbon degradation. Functional profiles were retrieved using get_subset_pathway() and visualized with plot_bubble() and plot_heatmap(). The HDP and PDP pathways were present in nearly all MAGs, except for a single *Sulfitobacter* genome recovered from the 700 m depth sample. On average, MAGs recovered 97% of the expected KO profiles for these pathways. In contrast, the NPP pathway was more taxonomically restricted. It showed strong representation in Gammaproteobacteria, partial presence in Alphaproteobacteria, and minimal detection in Flavobacteria. The genera *Glacieola* and the species *Marinobacter salarius* exhibited the highest representation of this pathway (Figure 3).

### Gene-level patterns

**FIGURE 4:**
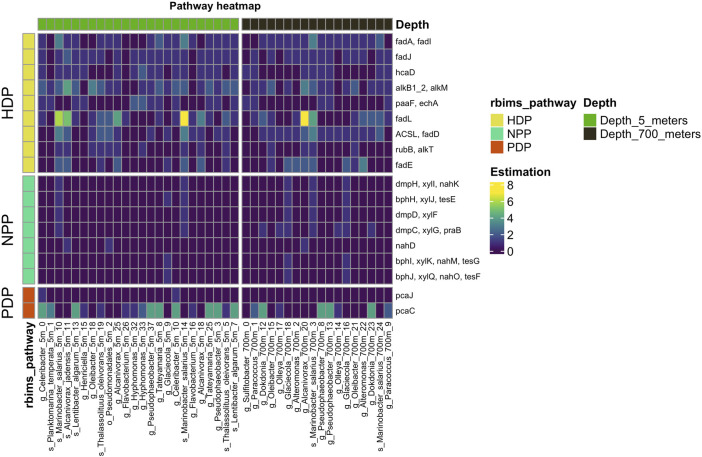
Heatmap of gene-level abundances in hydrocarbon degradation pathways. Abundance of individual genes associated with the HDP, PDP, and NPP across 42 MAGs. Rows correspond to genes and columns to MAGs. The color scale represents raw gene counts. The genes *pcaC* and *fadL* appear to be the most abundant and widely distributed.

To further examine gene-level distributions within these pathways, we used plot_heatmap() to visualize the abundance of individual KOs across MAGs. Several genes involved in aromatic hydrocarbon degradation were widely distributed across both shallow and deep communities. In particular, *pcaC* and *fadL* were among the most abundant genes detected across multiple taxa, suggesting a conserved role in the degradation of phenanthrene and hexadecane. In addition, genes involved in alkane degradation (*alkB*, *alkM*, and *alkT*) were detected across multiple genomes, supporting the presence of alkane-oxidizing capabilities in these microbial communities (Figure 4).

### Discriminant analysis

To investigate functional differentiation between shallow (5 m) and deep (700 m) communities, we applied the discriminant framework implemented in get_subset_discriminant(). This analysis prioritizes KOs that contribute to group separation based primarily on compositional effect-size estimates, with random forest used as an exploratory feature-ranking step (Supplementary Table S2).

**FIGURE 5:**
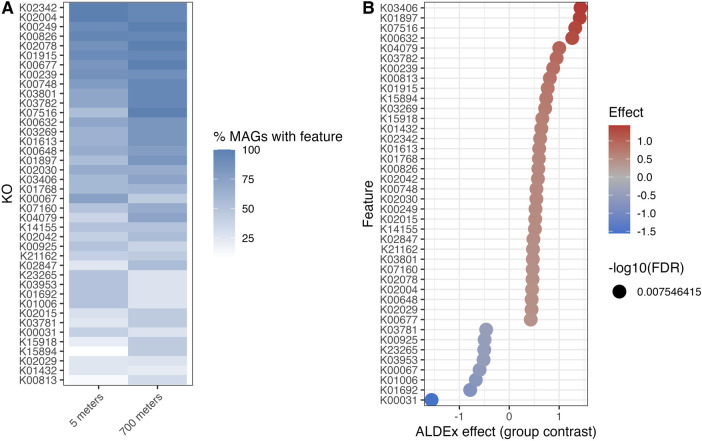
Output of the discriminant analysis. **(A)** Top 20 KOs selected from the discriminant analysis and their coverage per sampling environment. **(B)** ALDEx2 effect-size estimates, showing that the top KOs are enriched in MAGs from deep-water environments (effect >1).

At the gene level, several KOs showed strong directional enrichment toward deep-water MAGs, with effect sizes exceeding one in CLR-transformed space. Among the top-ranked features based on ALDEx2 effect sizes were K01897 (long-chain fatty-acid CoA ligase), K07516 (3-hydroxyacyl-CoA dehydrogenase), and K00382 (dihydrolipoamide dehydrogenase). These enzymes participate in fatty acid activation and redox metabolism, processes central to hydrocarbon degradation and downstream carbon assimilation (Figure 5B). The corresponding heatmap confirmed their consistent presence across deep-water genomes (Figure 5A).

To evaluate the stability of the exploratory random forest feature-ranking step, we repeated the analysis 200 times using stratified resampling of 80% of MAGs within each depth group. After filtering low-prevalence and zero-variance features, the analysis included 42 MAGs and 3,365 KOs. For each iteration, we recorded the top 20 KOs ranked by MeanDecreaseGini importance (Supplementary Table S3). K03778 was recovered among the top 20 features in 98.5% of iterations, and seven KOs appeared among the top 20 features in at least 50% of iterations (Supplementary Table S4). The mean pairwise Jaccard similarity among top-20 feature sets was 0.183, indicating that the complete random forest ranking varied across resampling iterations, while a subset of candidate KOs was repeatedly recovered (Supplementary Table S5). We therefore interpret the random forest output as exploratory feature prioritization rather than as evidence of predictive model performance.

### Pathway-level directional bias

**FIGURE 6:**
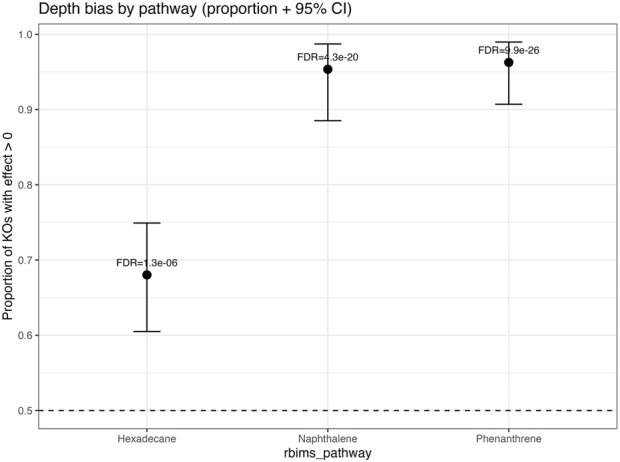
Pathway-level directional bias across hydrocarbon degradation pathways. Proportion of KEGG Orthologs (KOs) within each pathway showing positive discriminant effect values (effect >0) toward deep-water MAGs (700 m). Points represent the observed proportion of KOs with positive effect values within each pathway, and vertical bars indicate the 95% Clopper–Pearson confidence intervals. The dashed horizontal line at 0.5 represents the null expectation of no directional bias (i.e., equal probabilities of positive and negative effects). False discovery rate (FDR)-corrected p-values from exact binomial tests are shown above each pathway. The results reveal a strong directional enrichment toward deep-water communities for the phenanthrene and naphthalene degradation pathways, and a moderate but significant bias for the hexadecane degradation pathway.

While gene-level signals were informative, pathway-level aggregation revealed an even stronger pattern. When KOs were grouped according to curated hydrocarbon degradation pathways, we observed a highly consistent directional bias toward deep-water communities. In the phenanthrene degradation pathway, 103 out of 107 KOs exhibited positive effect values (prop = 0.96; FDR <1 × 10^−25^). Similarly, the naphthalene pathway showed 82 out of 86 KOs enriched toward depth (prop = 0.95; FDR <1 × 10^−19^), while the hexadecane pathway displayed 117 out of 172 KOs with positive effect values (prop = 0.68; FDR <1 × 10^−6^) (Supplementary Table S6). These results indicate that enrichment was not limited to isolated genes but instead reflected structurally coherent shifts across entire metabolic routes (Figure 6). Notably, this pathway-level directional consistency was not fully captured by coverage metrics alone (Figure 3), highlighting the added value of integrating gene-level discrimination with pathway-level aggregation.

### Protein family analysis

**FIGURE 7:**
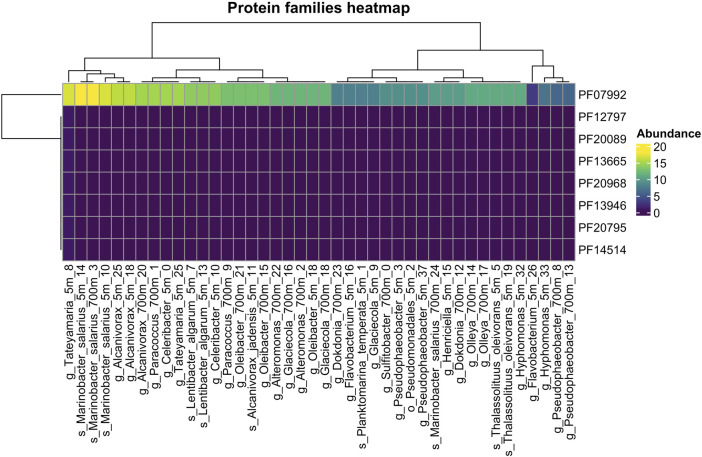
Heatmap of enriched domain abundances. Heatmap displaying the abundance of eight oxidoreductase domains across all MAGs. Dendrograms reflect clustering of MAGs (columns) and domains (rows) based on abundance profiles. PF07992 is abundant in the whole community with higher values present in the species *Marinobacter salarius* and the genus *Tateyamaria*. The latter also shows small abundances of the rest of the PFAMs profile.

To complement the pathway-level analysis, we explored protein family composition using get_subset_pca(), which identifies the most contributing Pfam domains along the principal component axes. This analysis revealed eight dominant oxidoreductase families, including NAD(P)H-dependent enzymes (PF07992, PF13665) and GMC oxidoreductases associated with alcohol and alkane oxidation (PF20089, PF12797). Visualization with plot_heatmap() showed that PF07992 was the most abundant domain across both shallow and deep MAGs. The highest abundances were observed in *Marinobacter salarius*, *Alcanivorax*, and *Tateyamaria*. These oxidoreductase domains support oxidative metabolism and NAD(P)H-dependent electron transfer, processes central to hydrocarbon degradation pathways (Figure 7).

Together, these analyses reveal a consistent functional structure underlying hydrocarbon degradation in the analyzed MAGs. By combining pathway coverage, gene-level abundance patterns, discriminant feature selection, and protein domain enrichment, *rbims* enabled the identification of both dominant metabolic strategies and lineage-specific functional traits involved in hydrocarbon degradation.

## Discussion

The application of the *rbims* workflow enabled the identification and comparison of hydrocarbon degradation pathways across 42 metagenome-assembled genomes (MAGs), providing a multi-layered view of functional potential at the level of KEGG orthologs, genes, and protein families. The analysis revealed consistent patterns across taxonomic groups that align with previous studies on hydrocarbon-degrading microbial communities.

In particular, the phenanthrene degradation pathway was more abundant in Alphaproteobacteria and Flavobacteriia lineages, consistent with previous enrichment studies of polycyclic aromatic hydrocarbon degraders (Tang et al., 2023; Yuan, 2015). Similarly, the hexadecane degradation pathway was strongly represented in Alphaproteobacteria and Gammaproteobacteria (Figures 3, 4). This pattern can be explained by the high abundance of *alkB* genes, which encode alkane monooxygenases responsible for the initial oxidation step in alkane degradation. Previous DNA-SIP experiments conducted with the same dataset reported multiple copies of *alkB* genes in several MAGs, suggesting a strong metabolic potential for alkane degradation in these communities (Vázquez Rosas Landa et al., 2023).

Several genes were consistently detected across multiple taxa, including *pcaC* and *fadL*, further supporting the presence of phenanthrene and hexadecane degradation capabilities in the analyzed MAGs. The *fadL* gene encodes an outer-membrane transporter involved in the uptake of long-chain fatty acids and hydrophobic substrates, including aliphatic and aromatic hydrocarbons, thereby facilitating their entry into bacterial catabolic pathways (Liu et al., 2022). Similarly, *pcaC* plays a central role in the protocatechuate branch of the β-ketoadipate pathway, which converts aromatic intermediates into compounds that feed into central carbon metabolism (Vidal-Silva et al., 2025). In addition, the widespread detection of the PF07992 domain, corresponding to rubredoxin-NAD(+) reductase (AlkT), further supports the presence of enzymatic machinery required for aerobic alkane degradation in both shallow and deep communities.

While these exploratory analyses confirmed the presence of hydrocarbon degradation pathways across the MAGs, the discriminant framework implemented in *rbims* revealed additional structure in the functional landscape. Gene-level discriminant analysis identified several KEGG orthologs enriched in deep-water MAGs, including K01897 (acyl-CoA synthetase), K07516 (acyl-CoA dehydrogenase), and K00382 (dihydrolipoamide dehydrogenase). These enzymes participate in fatty-acid activation, β-oxidation, and redox metabolism, processes that are fundamental to hydrocarbon catabolism.

The random forest component of rbims should be interpreted as an exploratory feature-ranking procedure rather than as a validated predictive model. In this study, it was used to prioritize candidate KOs for downstream biological interpretation, while the main inferential support came from ALDEx2 effect-size estimates and pathway-level directional bias testing. Although the stability analysis showed that a subset of candidate KOs was repeatedly recovered across resampling iterations, the complete top-20 ranking varied among iterations. Future applications aimed at prediction or classification should include formal model validation, including cross-validation, independent test sets, performance metrics, and additional feature-ranking stability analyses.

More importantly, aggregation of discriminant signals at the pathway level revealed a striking directional bias toward deep-water communities. The majority of KOs in the phenanthrene and naphthalene degradation pathways exhibited positive discriminant effects in the 700 m environment, indicating that enrichment was not limited to isolated genes but rather involved coherent shifts across entire metabolic routes. This pattern highlights the advantage of integrating gene-level discrimination with pathway-level aggregation, as the directional bias analysis detected functional differences that were not fully captured by coverage-based analyses alone. This differs from standard pathway enrichment approaches by emphasizing the direction and consistency of gene-level effects within a pathway, rather than only the number of significant features assigned to that pathway.

The analyzed community originates from samples collected in the Faroe–Shetland Channel, a deep-water region of the northeast Atlantic Ocean with a history of hydrocarbon exploration. Although no major oil seepage events have been reported in this area, the presence of these metabolic pathways suggests that microbial communities in this environment maintain a latent capacity for hydrocarbon degradation. Such metabolic potential may reflect long-term exposure to low hydrocarbon concentrations or the ecological versatility of marine heterotrophic bacteria in carbon-rich environments (Vázquez Rosas Landa et al., 2023)**.**

On another note, many software packages are available for genome annotation and metabolic profiling. To better position *rbims* relative to existing tools, we compared its functionality with related software, such as Thanos, MetQy, MetaPath, and MNet/mmmnet to account for differences in functional profiling, pathway exploration, and metabolic interpretation (Table 2) (Cao et al., 2015; Martinez-Vernon et al., 2018; Wang et al., 2012; Zhao et al., 2024). *rbims* is not intended to replace primary genome annotation tools and, unlike network-centered tools, *rbims* does not infer or model interaction graphs; instead, it provides a downstream framework for heterogeneous annotation import, metadata stratification, user-defined pathway exploration, ALDEx2-based discrimination analysis with random forest–based feature ranking, pathway-level directional bias testing, and exportable visualizations.

Beyond the biological insights, the *rbims* framework showed efficient performance for the datasets evaluated in this study. Processing a dataset of eight MAGs required approximately 1.5 GB of RAM, while the full dataset of 42 MAGs required approximately 3.2 GB of RAM, with peak usage around 5 GB. Execution on a high-performance computing (HPC) cluster reduced runtime by approximately 59% for the smaller dataset and up to 84% for the 42-MAG dataset. These results indicate that *rbims* can run efficiently on standard workstations for small-to medium-scale MAG datasets and can benefit from HPC execution. However, larger benchmarks involving hundreds or thousands of MAGs will be needed to fully evaluate scalability across very large genome-resolved metagenomic collections.

The most computationally demanding steps occur upstream of *rbims*, during functional annotation with tools such as KofamScan, dbCAN, InterProScan, and MEROPS, which require large and frequently updated reference databases. Once these annotations are generated, *rbims* operates mainly on parsed annotation tables and standardized data frames, reducing the computational burden of downstream exploration, visualization, and statistical analysis. To improve reproducibility across upstream tools, *rbims* provides a preconfigured Conda environment that integrates specific versions of these annotation programs and their corresponding databases. Although the environment is optional, its use simplifies installation, ensures version consistency, and facilitates integration with HPC infrastructures.

## Conclusion

The *rbims* package provides a flexible and modular framework for processing, quantifying, and visualizing functional annotations derived from genome-resolved metagenomic datasets. By integrating outputs from multiple annotation databases, including KEGG, InterProScan, dbCAN, MEROPS, and Picrust2, *rbims* enables researchers to explore metabolic traits across microbial genomes while incorporating experimental metadata for comparative analyses.

In this study, *rbims* facilitated the identification of hydrocarbon degradation pathways across 42 MAGs and revealed consistent enrichment patterns in deep-water communities. The integration of exploratory analyses, discriminant feature selection, and pathway-level aggregation enabled us to detect functional shifts not fully captured by traditional coverage-based approaches.

Although *rbims* was originally designed for genome-resolved metagenomics, its modular structure also makes it adaptable to multi-omic studies. Gene and pathway-level outputs generated by *rbims* can be combined with metatranscriptomic or metaproteomic datasets to investigate functional activity, validate predicted metabolic capabilities, or guide experimental design. This flexibility positions *rbims* as a useful tool for hypothesis generation and ecological interpretation in microbial community research.

## Data Availability

The original contributions presented in the study are included in the article/Supplementary Material, further inquiries can be directed to the corresponding author.
